# Supplementary material for: The Genomic Landscape of Centromeres in Cancers
Source: Sci Rep. 2019 Aug 2;9:11259. doi: 10.1038/s41598-019-47757-6 (PMC6677800; doi:10.1038/s41598-019-47757-6)
Supplement: Supplementary file 1 — Supplementary information [file 41598_2019_47757_MOESM1_ESM.docx]

The Genomic Landscape of Centromeres in Cancers

Anjan K. Saha, Mohamad Mourad, Mark H. Kaplan, Ilana Chefetz, Sami N. Malek, Ronald Buckanovich, David M. Markovitz, and Rafael Contreras-Galindo

**SUPPLEMENTARY FIGURES AND LEGENDS**
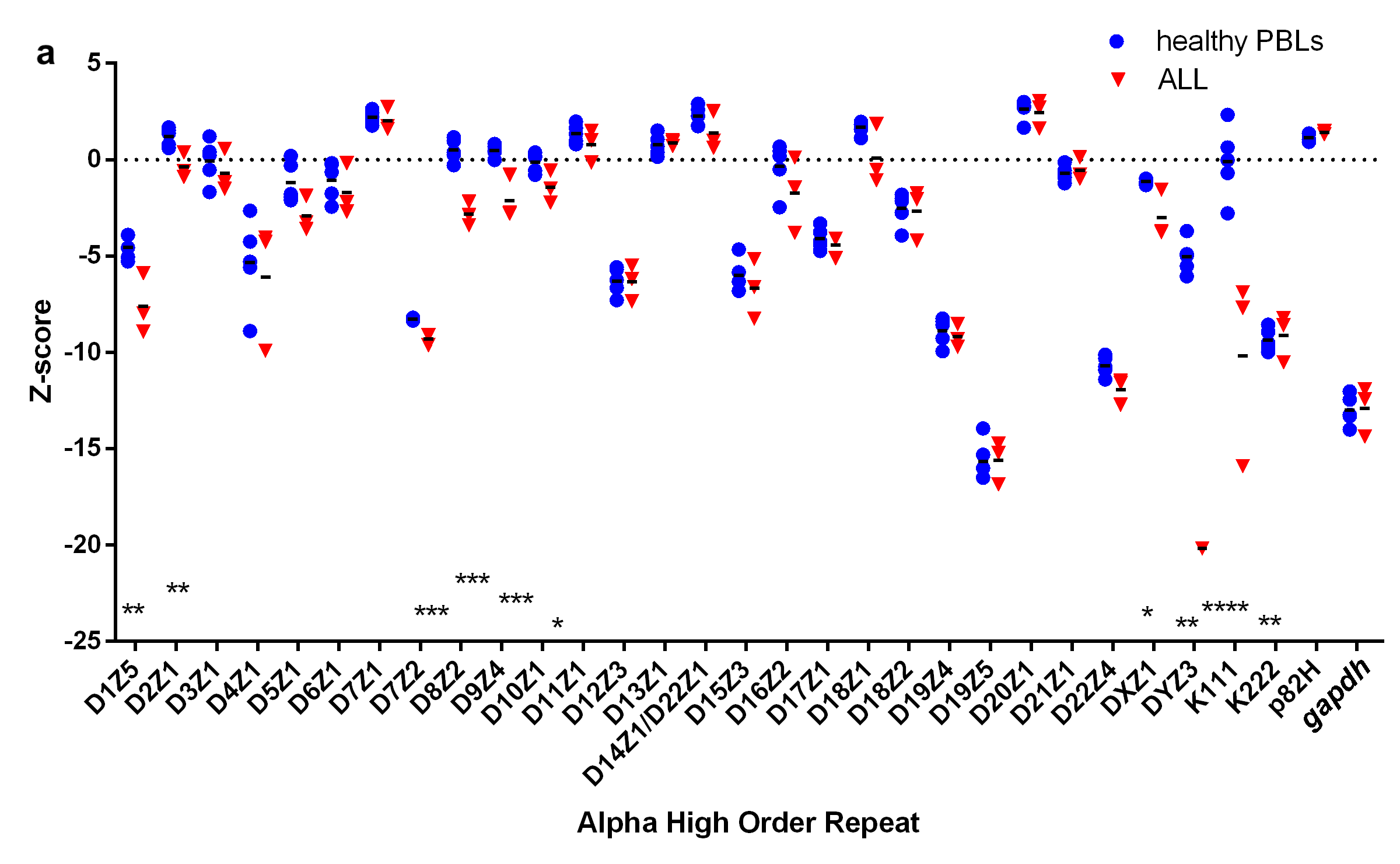


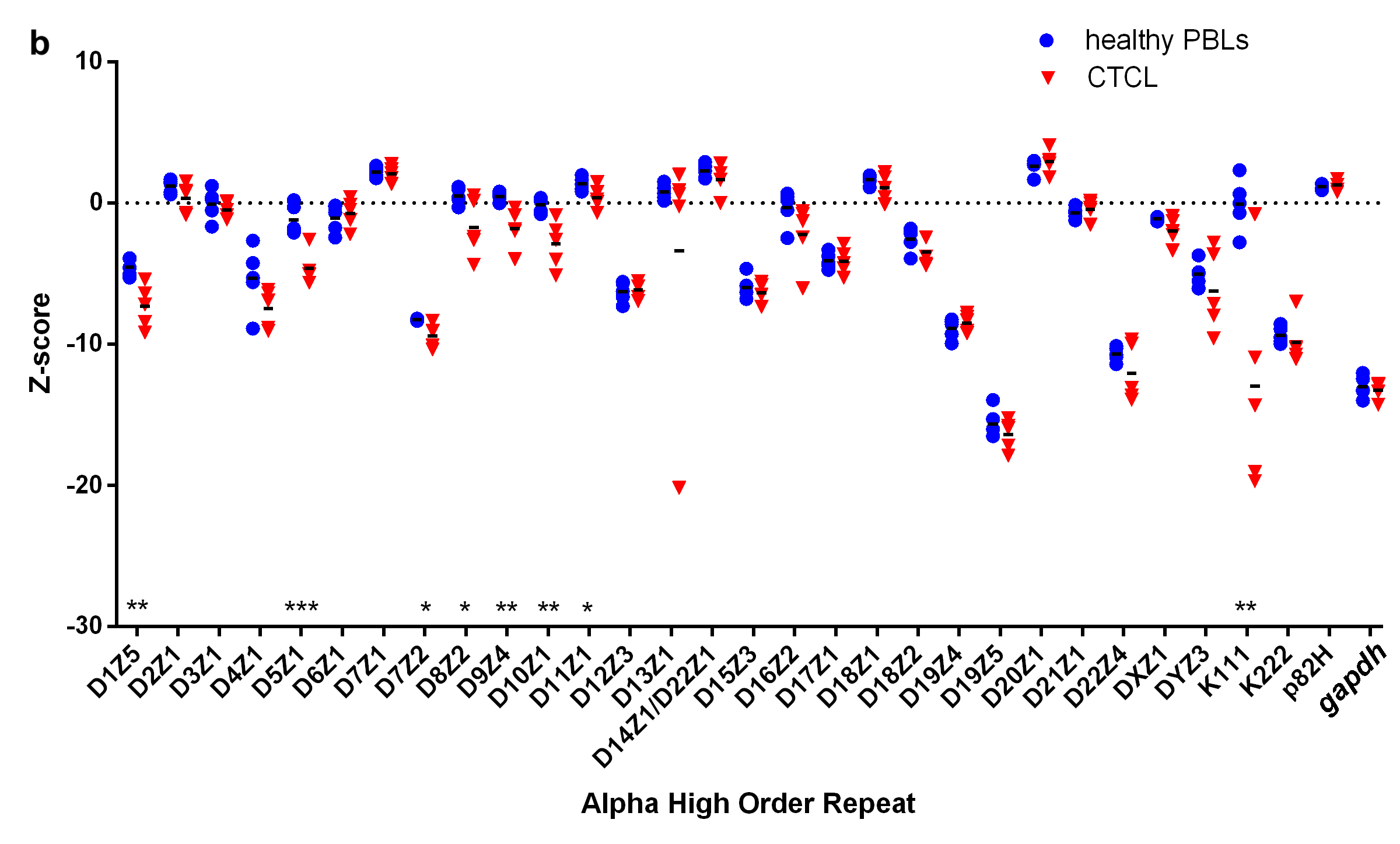


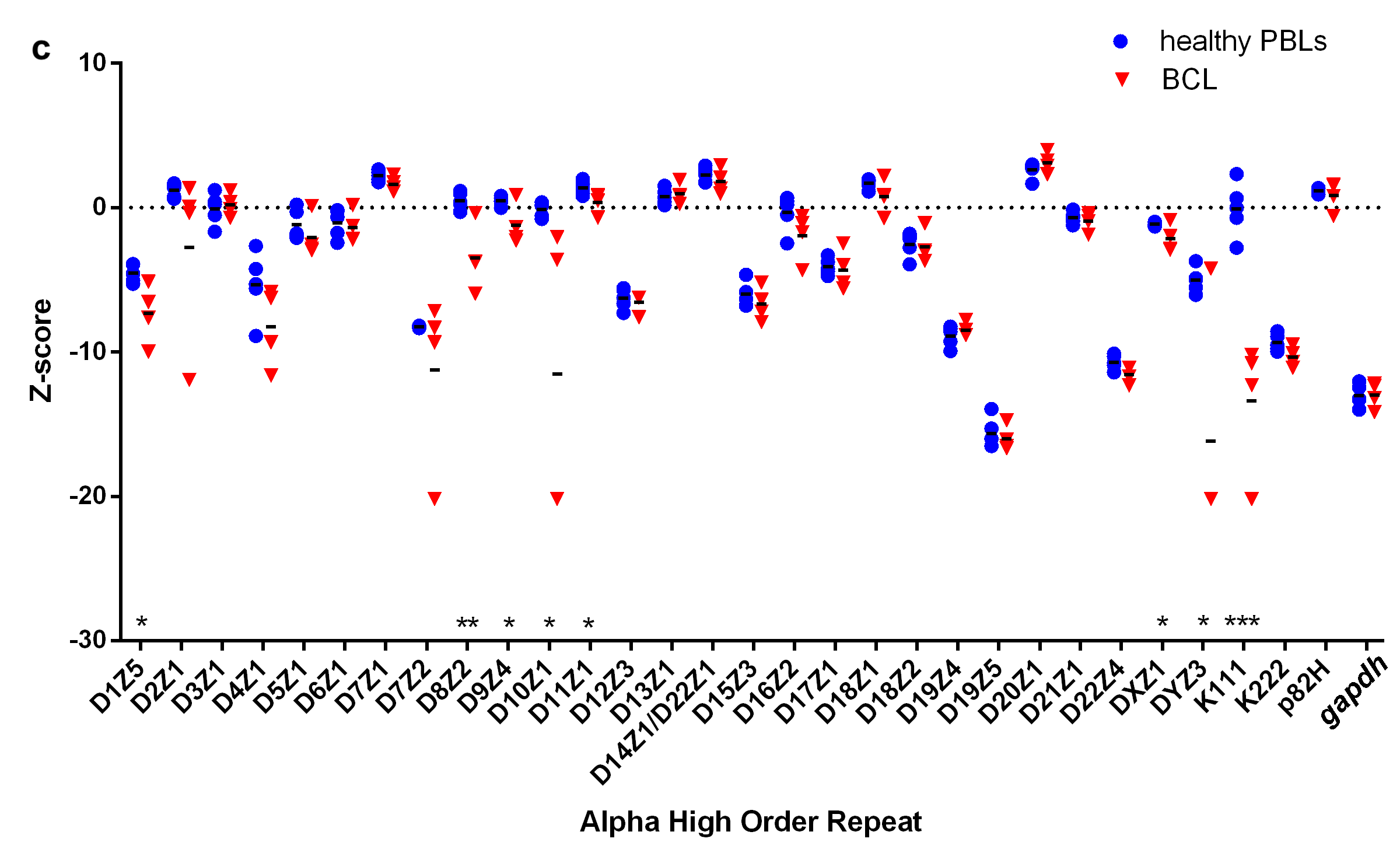


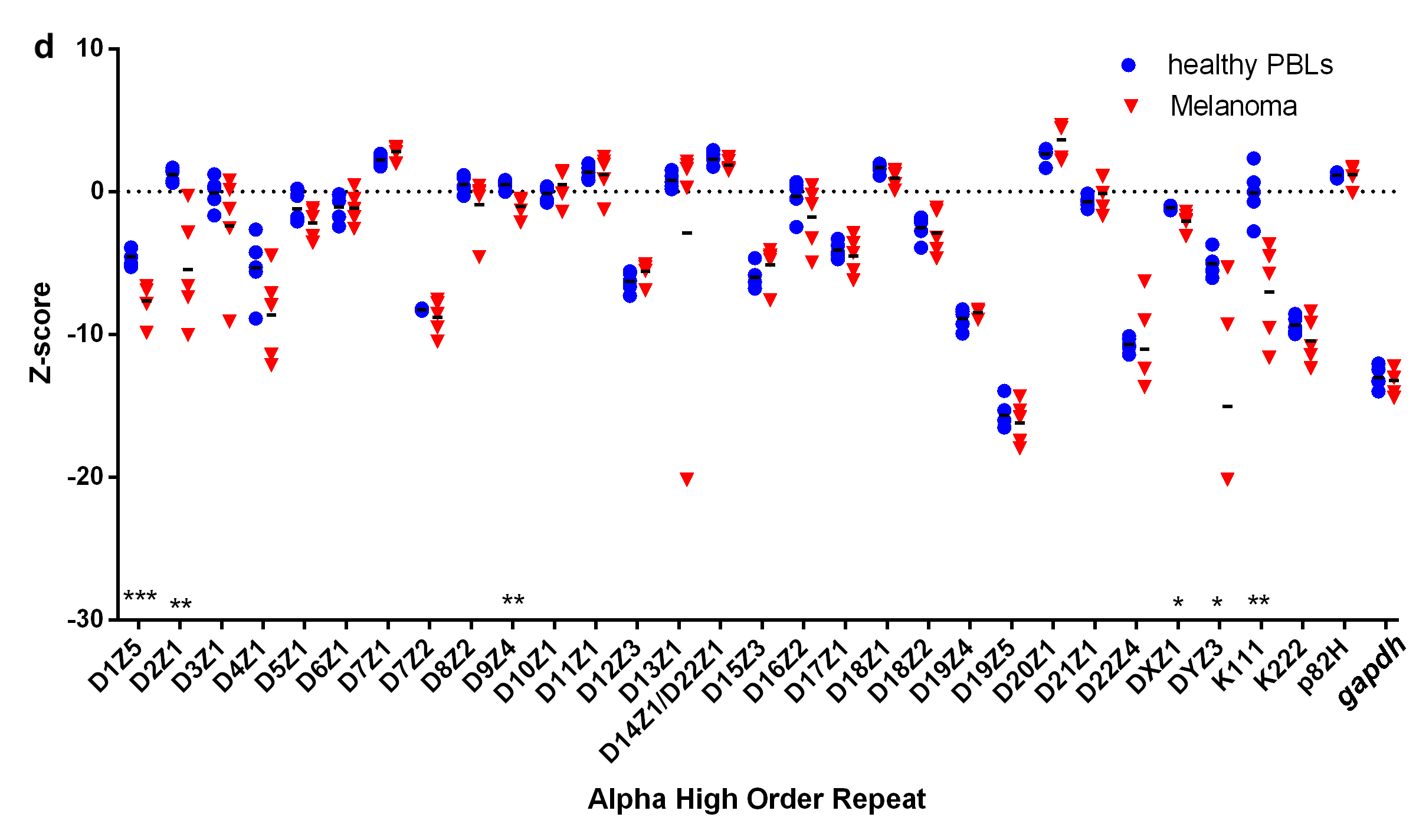


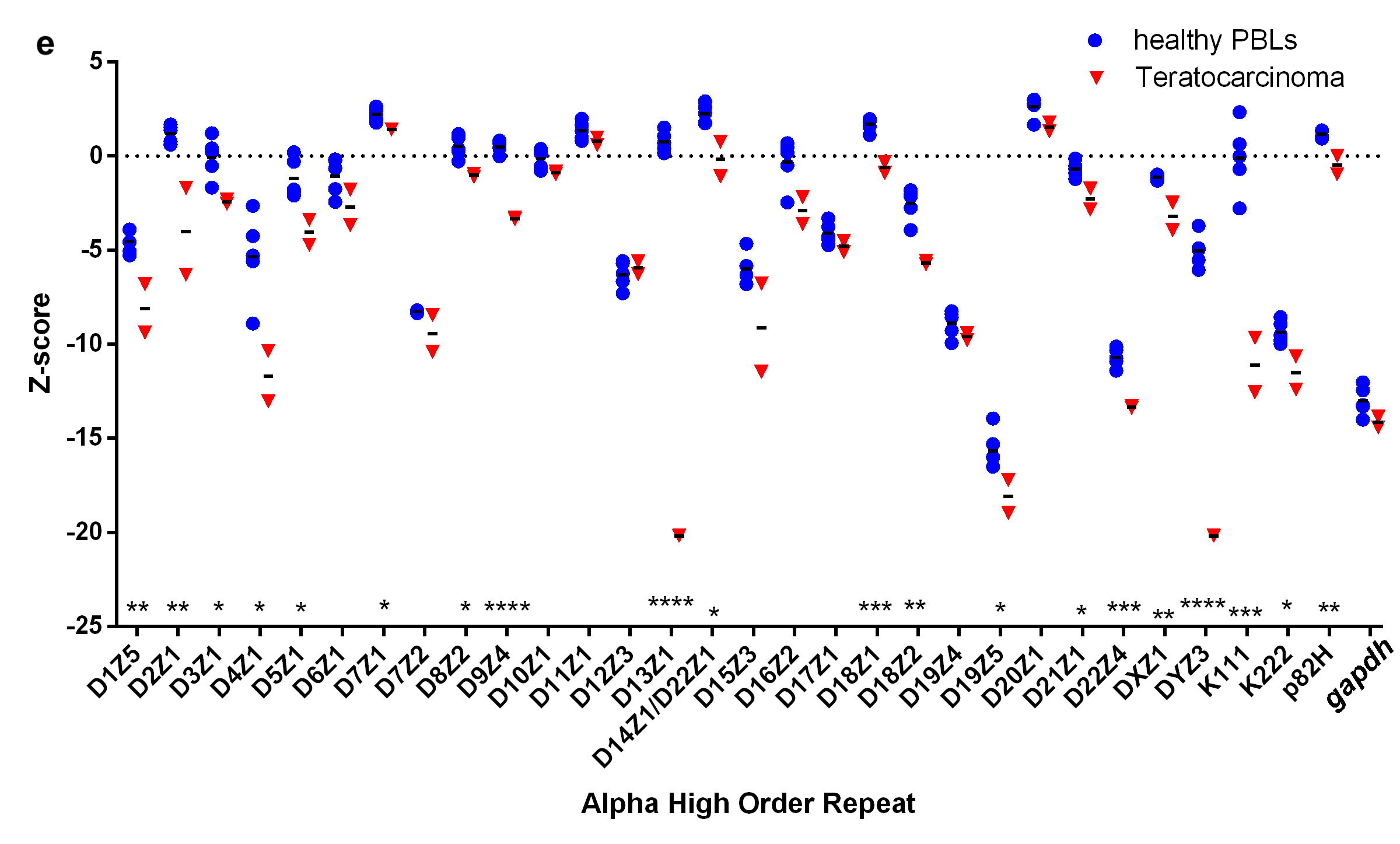
**Supplementary Figure S1.** Heterogeneous loss of centromeres in cancer cell lines. Dot plots representing the abundance of α-satellites specific for each centromere array (X axis) in (**a**) Acute Lymphoblastic Leukemia (ALL) cell lines, (**b**) Cutaneous T-Cell Lymphoma (CTCL) cell lines, (**c**) B-Cell Lymphoma (BCL) cell lines, (**d**) melanoma cell lines, and (**e**) teratocarcinoma cell lines. Each dot represents a DNA sample from a distinct cell line. Abundance of centromere specific α-satellites is depicted by the Z-score (Y-axis) of each α-satellite. The Z-scores show the log number of standard deviation differences to the mean of the whole data set in order to appreciate the difference in the number of repeats in each centromere array. The log_2_ normalized numbers for each α-satellite were normalized to the average copy number of repeats in healthy cells (blue circles). Statistical significance was calculated using a t-test. * = *p*< 0.05, ** = *p*< 0.01, *** = *p*<0.001, **** = *p*< 0.0001.


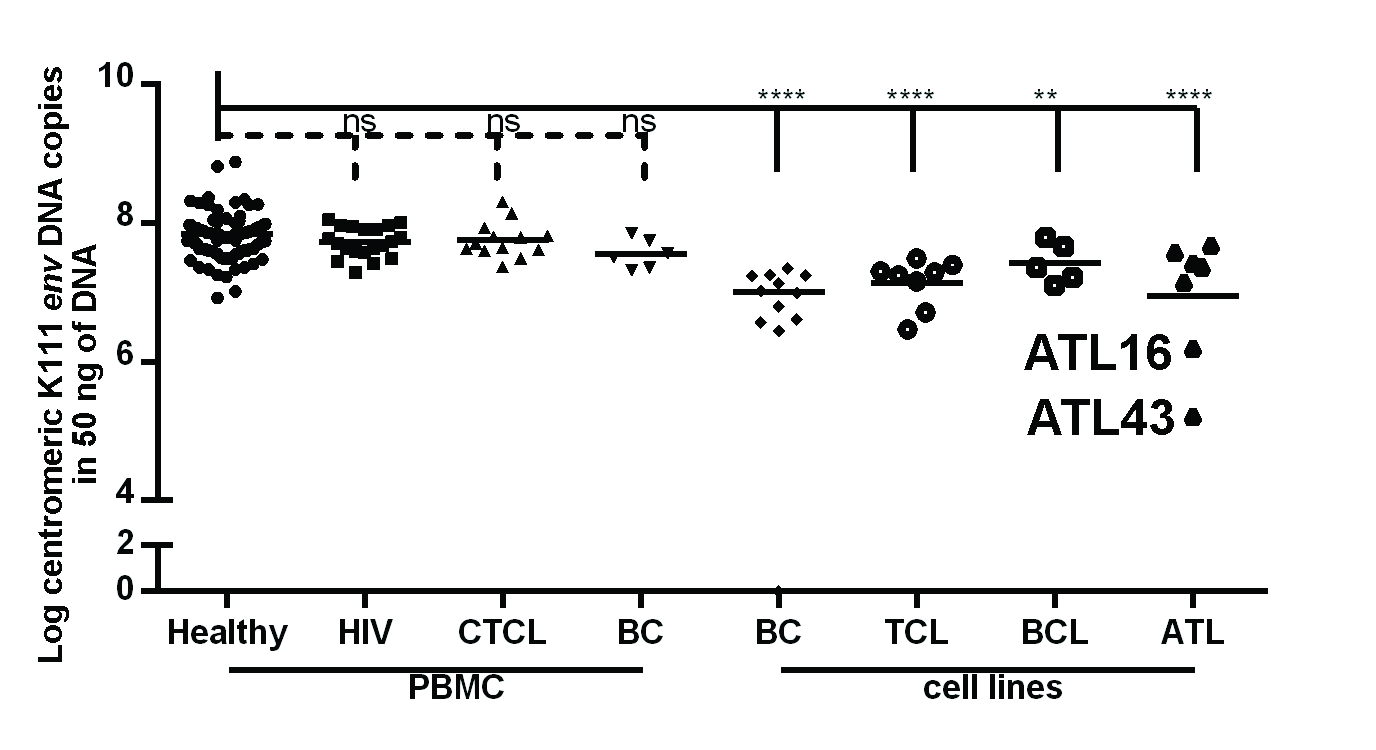


**Supplementary Figure S2.** Abundance of pericentromeric K111 across cell lines and PBMCs. Dot plot representing log-transformed abundance of centromeric HERV *env* sequences from either PBMCs or cell lines with disease type designations listed along the X axis. The *gag* region of K111 was not assessed in our analysis, as select populations and CTCL patients are homozygous null for K111 *gag* (Kaplan et. al. *BMC Medical Genomics*, in press). HIV = HIV patient sample, CTCL = Cutaneous T-Cell Lymphoma, BC = Breast Cancer, TCL = T-Cell Lymphoma, BCL = B-Cell Lymphoma, ATL = Adult T-Cell Lymphoma. Abundance of K111 is depicted by the log_2_ Z-score (Y-axis). Statistical significance was calculated using a t-test. * = *p*< 0.05, ** = *p*< 0.01, *** = *p*<0.001, **** = *p*< 0.0001.


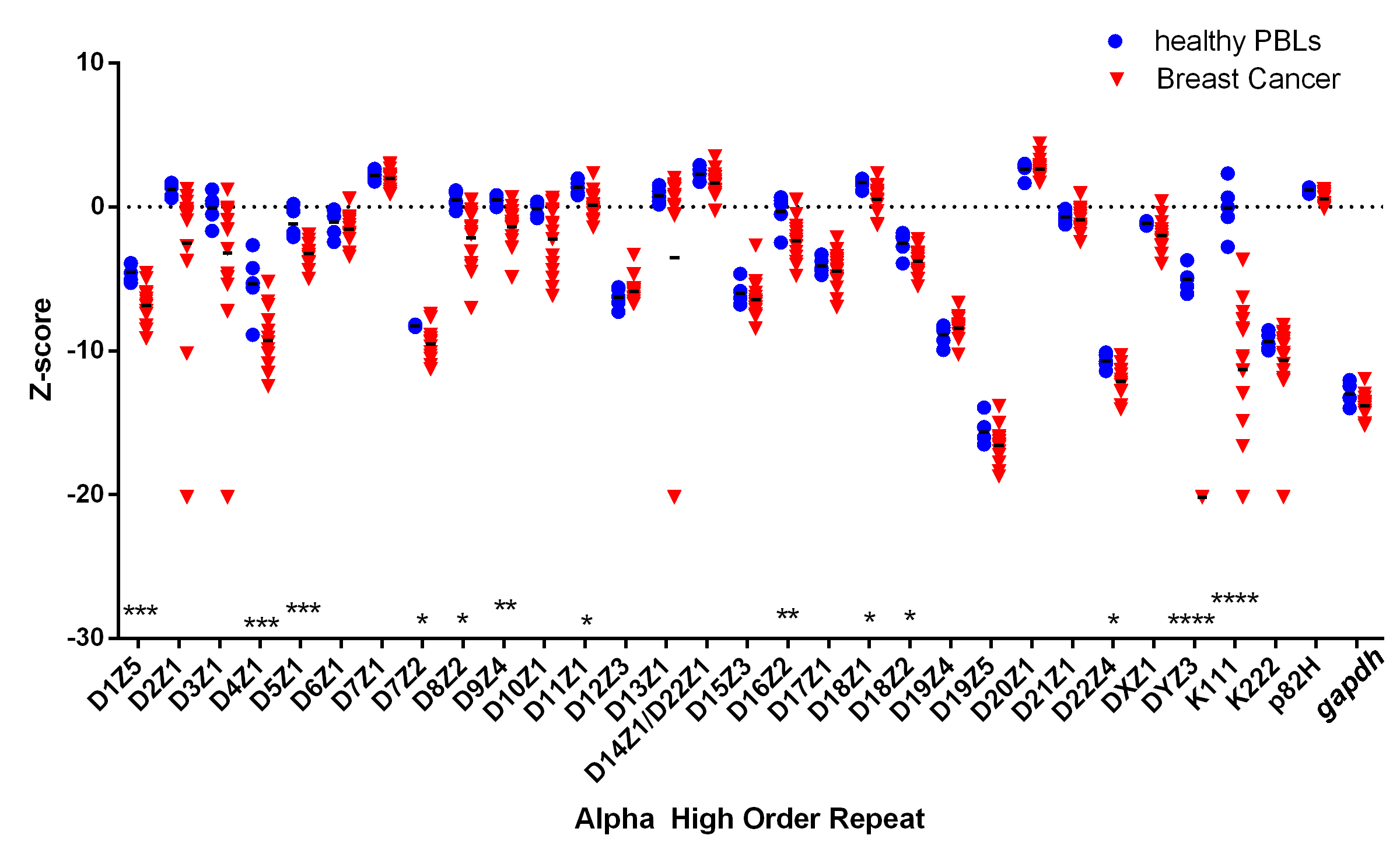


**Supplementary Figure S3.** Heterogeneous loss of centromeres in breast cancer cell lines. Dot plot representing the abundance of α-satellites specific for each centromere array (X axis) in breast cancer cell lines. Each dot represents a DNA sample from a distinct cell line. Abundance of centromere specific α-satellites is depicted by the Z-score (Y-axis) of each α-satellite. The Z-scores show the log number of standard deviation differences to the mean of the whole data set in order to appreciate the difference in the number of repeats in each centromere array. The log_2_ normalized numbers for each α-satellite were normalized to the average copy number of repeats in healthy cells (blue circles). Statistical significance was calculated using a t-test. * = *p*< 0.05, ** = *p*< 0.01, *** = *p*<0.001, **** = *p*< 0.0001.


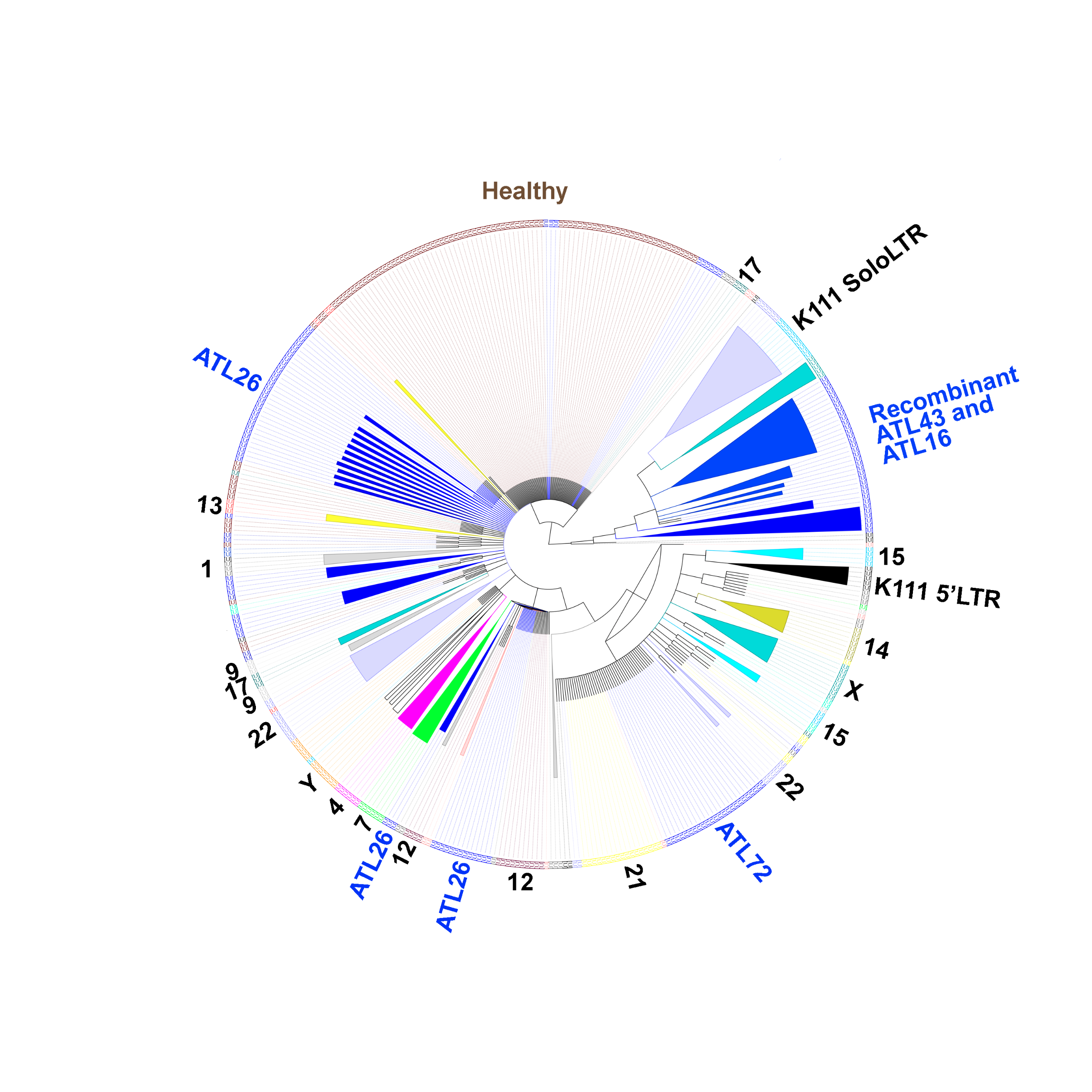


**Supplementary Figure S4.** Gene conversion of HERV K111 in adult T-cell leukemia (ATL) cell lines. Phylogenetic analysis conducted on K111 sequences amplified by PCR in ATL cell lines (ATL26, ATL72, ATL16, and ATL43), human-hamster hybrid cell lines, and heathy cells (brown labels). Amplicons are labeled and color-coded along the edge of the phylogenetic tree according to the cell line that produced the amplicon. Amplicons from human-hamster hybrid cell lines are denoted numerically by the human chromosome present in each hybrid cell line. Amplicons from K111 5’LTR and Solo LTR are additionally denoted. Recombinant K111 sequences resembling Solo LTRs are seen in cell lines ATL43 and ATL16, shown in blue arising from the same ancestral sequence to K111 Solo LTR sequences. K111 sequences from heathy PBLs show heterogeneous distribution along the tree and did not cluster in novel clades.





**Supplementary Figure S5.** Heterogeneous loss of centromeres in ovarian cancer patients. Dot plot representing the abundance of α-satellites specific for each centromere array (X axis) in ovarian cancer tumors. Each dot represents a DNA sample from a distinct cell line. Abundance of centromere specific α-satellites is depicted by the log_2_ Z-score (Y-axis) of each α-satellite. The log_2_ normalized numbers for each α-satellite were normalized to the average copy number of a given repeat in DNA from healthy cells (blue circles). Statistical significance was calculated using a t-test. * = *p*< 0.05, ** = *p*< 0.01, *** = *p*<0.001, **** = *p*< 0.0001.





**Supplementary Figure S6.** Heterogeneous loss of centromeres in CLL patients. Dot plot representing the abundance of α-satellites specific for each centromere array (X axis) in CLL tumors. Each dot represents a DNA sample from a distinct cell line. Abundance of centromere specific α-satellites is depicted by the log_2_ Z-score (Y-axis) of each α-satellite. The log_2_ normalized numbers for each α-satellite were normalized to the average copy number of a given repeat in DNA from healthy cells (blue circles). Statistical significance was calculated using a t-test. * = *p*< 0.05, ** = *p*< 0.01, *** = *p*<0.001, **** = *p*< 0.0001.
